# Supplementary material for: Is it possible to reconstruct an accurate cell lineage using CRISPR recorders?
Source: eLife. 2019 Jan 28;8:e40292. doi: 10.7554/eLife.40292 (PMC6349403; doi:10.7554/eLife.40292)
Supplement: Transparent reporting form [file elife-40292-transrepform.docx]

***eLife’s* transparent reporting form**

We encourage authors to provide detailed information *within their submission* to facilitate the interpretation and replication of experiments. Authors can upload supporting documentation to indicate the use of appropriate reporting guidelines for health-related research (see [EQUATOR Network](http://www.equator-network.org/ )), life science research (see the [BioSharing Information Resource](https://biosharing.org/)), or the [ARRIVE guidelines](http://www.plosbiology.org/article/info:doi/10.1371/journal.pbio.1000412) for reporting work involving animal research. Where applicable, authors should refer to any relevant reporting standards documents in this form.

If you have any questions, please consult our Journal Policies and/or contact us: [editorial@elifesciences.org](mailto:editorial@elifesciences.org).

**Sample-size estimation**

- You should state whether an appropriate sample size was computed when the study was being designed
- You should state the statistical method of sample size computation and any required assumptions
- If no explicit power analysis was used, you should describe how you decided what sample (replicate) size (number) to use

Please outline where this information can be found within the submission (e.g., sections or figure legends), or explain why this information doesn’t apply to your submission:

For the main part of our *in silico* simulation experiments, the reported statistic is the accuracy of lineage reconstruction estimated using a published algorithm (Robinson-Foulds (RF)) which is expressed as a percentage. The number we report represents the mean RF accuracy score of 1,000 computer simulations (as in Figures 5, 6, 7 and 9).

For simulations where we were more intensively exploring the parameter space (different mutation rates and/or number of targets) the reported accuracy represents the mean RF accuracy score of 10 computer simulations (Figures 2,and 7).

The number of simulations used for each experiment is stated in the Methods and Materials section.

**Replicates**

- You should report how often each experiment was performed
- You should include a definition of biological versus technical replication
- The data obtained should be provided and sufficient information should be provided to indicate the number of independent biological and/or technical replicates
- If you encountered any outliers, you should describe how these were handled
- Criteria for exclusion/inclusion of data should be clearly stated
- High-throughput sequence data should be uploaded before submission, with a private link for reviewers provided (these are available from both GEO and ArrayExpress)

Please outline where this information can be found within the submission (e.g., sections or figure legends), or explain why this information doesn’t apply to your submission:

The sequence data stemming from our sequencing experiment derives from a multiplexed sequencing run of 6 pools of 10-100 individuals. Here we are sequencing the PCR products amplified from up to tens of thousands of cells per individual and different cells may have different products. We did not use technical or biological replicates and this experimental design is reported in the methods.

Raw data from the sequencing run and related reports, as well as quality-trimmed reads separated by condition are available at:

https://datadryad.org/review?doi=doi:10.5061/dryad.qb7r0d3

doi:10.5061/dryad.qb7r0d3

**Statistical reporting**

- Statistical analysis methods should be described and justified
- Raw data should be presented in figures whenever informative to do so (typically when N per group is less than 10)
- For each experiment, you should identify the statistical tests used, exact values of N, definitions of center, methods of multiple test correction, and dispersion and precision measures (e.g., mean, median, SD, SEM, confidence intervals; and, for the major substantive results, a measure of effect size (e.g., Pearson's r, Cohen's d)
- Report exact p-values wherever possible alongside the summary statistics and 95% confidence intervals. These should be reported for all key questions and not only when the p-value is less than 0.05.

Please outline where this information can be found within the submission (e.g., sections or figure legends), or explain why this information doesn’t apply to your submission:

For all results coming from each set of simulations, we report the cell lineage reconstruction accuracy as:

Either 1) mean accuracy for different mutation rates and number of targets (represented as heatmaps) (figures 2 and 7)

Or 2) mean accuracy values showing the 95% confidence intervals (Figure 2)

Or 3) the distribution of all simulations as violin or boxplots (plots 5-9)

In each case, the number of simulations and samples used are explicitly stated in the main text in the Methods and Materials section.

(For large datasets, or papers with a very large number of statistical tests, you may upload a single table file with tests, Ns, etc., with reference to sections in the manuscript.)

**Group allocation**

- Indicate how samples were allocated into experimental groups (in the case of clinical studies, please specify allocation to treatment method); if randomization was used, please also state if restricted randomization was applied
- Indicate if masking was used during group allocation, data collection and/or data analysis

Please outline where this information can be found within the submission (e.g., sections or figure legends), or explain why this information doesn’t apply to your submission:

This does not apply to our study as we did not perform group allocation.

**Additional data files (“source data”)**

- We encourage you to upload relevant additional data files, such as numerical data that are represented as a graph in a figure, or as a summary table
- Where provided, these should be in the most useful format, and they can be uploaded as “Source data” files linked to a main figure or table
- Include model definition files including the full list of parameters used
- Include code used for data analysis (e.g., R, MatLab)
- Avoid stating that data files are “available upon request”

Please indicate the figures or tables for which source data files have been provided:

In the Github repository “<https://github.com/irepansalvador/CRISPR_recorders_sims>” (doi: <https://doi.org/10.5281/zenodo.1320791>) we provide the following scripts:

For Figures 2-3, and 5-7:

MATLAB scripts for the simulations.

Associated with figures 3 and 4:

- Jupyter notebooks (Python and R scripts) used for the analysis of sequencing reads and for estimating the mutation rate of the FAST target.

For re-creating heatmap in Figure 7:

- R scripts and data.

For Figures 2, 3, 5, 6, 7, Figure 2-figure supplement 1, Figure 5-figure supplement 1 and Figure 7-figure supplement 2:

- Perl scripts for making NEXUS files (to use as input in PAUP) from the simulations’ output.

For Figure 6 and Figure 6-figure supplement 1:

- Perl script to analyse the GESTALT v7 recorder.
